# Cholera—Absence of the Precursory Diarrhœa—Treatment of the Disease

**Published:** 1849-08

**Authors:** 


*Cholera—Absence of the Precursory Diarrhœa—Treatment of the Disease*—At a meeting of the Royal Medical and Chirurgical Society, (Feb. 27,) Mr. Streeter said that he "should like to direct attention to the fact of the very large number of cases of cholera which occur without premonitory diarrhœa, and to the greater danger attending those cases; for I do not think that sufficient attention has been paid to those cases in which collapse has been ushered in without previous symptoms, and to those in which there is scarcely any vomiting and purging, and which, I believe are universally fatal. There are a large number of cases in which the disease is ushered in without previous purging and vomiting. With reference to treatment, I think a distinction should be made between cases of collapse originating directly from the poison, and those in which premonitory diarrhœa has existed for several days, as in those cases the collapse is due partly to the poison, and partly to the exhaustion from serous evacuations. After the most careful observation, I have come to the belief that there can be no recovery except where nature or art sets up the action of vomiting; and, as to remedies, any attempt to check that process checks the mode which nature adopts to overcome the disease. We are constantly told, in reference to the progress of a patient, that all is well 'except the vomiting;' but I believe that the only successful way to produce reaction is to encourage the vomiting, and sustain it at intervals, regulated by the powers of the patient. With respect to the agent for the production of this, it seems to me to matter little; mustard and salt have been favorite remedies, and certainly the latter, from its known harmlessness, is worthy of adoption; cold water, also, will in many instances succeed; but there have been cases in which it will not keep it up, and then powerful stimuli must be employed."

Dr. Baly remarked that the subject of the relation of the precursory diarrhœa to cholera is important. The question is, whether the diarrhœa is a part of the disease, and if so, whether we can cut it short. "As far as I have observed at Millbank Prison, the diarrhœa is of three classes: in the one, there are one or two evacuations before collapse; in the second, there is serious diarrhœa, rice water evacuations, and occasional vomiting. In both these cases, I think the diarrhœa is a part of the disease; but it seems to me impossible to stop it by the ordinary remedies. Then there is the third kind, in which at first the evacuations have not the character of rice water, and there is no vomiting or cramp. This goes on for several days, and in the early stage may be checked. The choleraic diarrhœa subsequently ensues. It appears to me that the early diarrhœa in such cases, of which I have seen several, is not a part of the disease, but predisposes to it; but this class is not very frequent; in the more frequent cases the ordinary remedies fail in cure. As to treatment of the cholera itself, I have not met with any greater success than others, and I believe that no one will succeed till a specific be found for it. We are at greater disadvantage in cholera than in typhus fever, because the effects of the typhus poison are more under our control than those of the cholera poison. Strong stimulants, I think, are of little avail, and, generally, the less we do the better. Warmth is beneficial, but not a great degree of heat; a very moderate degree of heat is certainly good. I should also recommend the administration of cold water, not for the purpose of producing vomiting, but to dilute the blood. I have found benefit from the use of chloroform, which acts as a palliative, by relieving pain, the cramps, and vomiting."

Dr. Baly said, that he had used the injection of saline fluids into the veins in six cases, and the result was uniformly death. "In the first case, the effect of it was very encouraging; the patient previously appeared to me as if in his last gasp; in a quarter of an hour, he breathed gently, seemed in a quiet sleep, and the color in the cheek was natural. In no other case was the effect so marked: while in all there was a return to collapse. In two cases I employed large doses of calomel; one man had five 15-grain doses and five scruple doses, and after death it was all found in his stomach, with the exception of a small portion in the duodenum."

Mr. Streeter believes that the experience in Great Britain in the former epidemic has settled the point, that the treatment by injections of salines into the veins should be abandoned. "I at one time hoped much from stimulating the skin by mustard cataplasms on the arms and over the abdomen; they reddened the skin, but produced no reaction, and this was tried in several cases. I found that, unless vomiting was produced, reaction did not occur; and this from an experience that extended over some hundreds of cases. It is, I think, important that we should treat cases of diarrhœa as if they would turn to cholera, and my prescription generally was: 1 gr. opium, 1 gr. acetate of lead, 1 gr. capsicum, 1 gr. calomel. With respect to large doses of calomel, I found no benefit arising from them. (I gave half a drachm in one instance.) I also saw phosphorus administered in two cases, and in one case a pill was found, as taken, in the stomach, and, in the other, in the appendix vermiformis."

Mr. Busk, surgeon of the Dreadnought, stated that "out of forty cases that have come under my notice, about half have terminated fatally; so that, while the cases have not been numerous, they have been very fatal, and have been generally confined to young men of muscular strength, and who had been in a condition to obtain sufficient wholesome food; the majority of them have been from colliers. In these cases, I have observed a considerable difference in the type of the disease between that now prevailing and in the previous epidemic. I refer to the greater proportional number who have not died in a state of collapse at the present time; they have generally died in a state of oppression and coma, and almost invariably with suppression of the urinary secretion. Also, in the symptoms of the disease in the state of collapse there is considerable difference; the cramps are not so severe, the coldness not so intense, and the facility with which heat is restored is much greater now than formerly. In many cases the temperature in the axilla was 96, and that under the tongue 78, showing that the blood in the lungs was cool and probably stagnating. Another difference is, the absence of perspiration, while, in 1832, the perspiration was enormous. The duration of the disease, also, was very much longer, from their not dying in a state of collapse. On the treatment, I can add nothing to the observations of Dr. Baly and others; my opinion is, that we have no treatment, heroic or otherwise, suited to control the disease: the only thing I am certain of is, that chloroform relieves the cramps, and thus has a beneficial influence in the way of relief; but it has no other control over the disease. The chief force of the disease, at the present time, seems to fall on the kidneys; no means have had the effect of inducing secretion of the urine; turpentine epithems and frictions have alike been unsuccessful. Another point is that, after the urine has been suppressed, and when it is again passed, the first is invariably albuminous; the tubuli are usually found crammed with epithelium, and the functions of the gland are thus obstructed. The great thing here is, to excite the action of the kidneys. With regard to the pathology of the disease, I have nothing particular to offer. There is an affection, however, of the large intestine which I do not remember to have seen described. It usually occurs in the transverse arch of the colon, and consists at first of congestion of a patch of the mucous membrane, which, if the case is prolonged, seems to be followed by circumscribed gangrene. In these cases, especially, it would appear that bloody motions are passed; but motions of this character are not limited to them. Blood, in any quantity, in the motions, has almost invariably been a fatal sign. (The speaker here exhibited two specimens, consisting of the transverse arch of the colon, showing the simple ecchymosis and gangrene of the mucous membrane.) No doubt it must be very violent action which produces this extreme disorganization; the disease appears to depend upon the introduction of a morbid poison, which nature making an effort to eliminate, the attempt destroys the membrane.—*Med. Times.*

